# Occupational physical demands in eldercare workers: a systematic scoping review of studies reporting quantitative data

**DOI:** 10.1007/s00421-025-05962-4

**Published:** 2025-09-09

**Authors:** Nestor Lögdal, Jennie A. Jackson, Svend Erik Mathiassen, Sven Svensson, David M. Hallman

**Affiliations:** Department of Occupational Health, Psychology, and Sports Sciences, University of Gavle, Gävle, Sweden

**Keywords:** Physical workload, Physiological demands, Nursing home, Homecare, Temporary workers

## Abstract

**Aim:**

To summarize the literature on quantitative measures of physical demands in eldercare, with attention to differences between temporary and permanent workers, and to identify gaps to guide future physiological research.

**Methods:**

We searched Scopus, Web of Science, and PubMed for English and Swedish peer-reviewed studies on physical demands in eldercare. Risk of bias was assessed, and descriptive data extracted.

**Results:**

We identified 37 relevant articles where physical demands were assessed via self-report (*n* = 23), biomechanical modeling (*n* = 6), and direct measurement (*n* = 8). Risk of bias assessment showed generally insufficient descriptions of study settings and poor descriptions of instruments assessing physical demands. Workers reported physical demands ranging from 40 to 98% maximum (different scales across studies). Biomechanical models showed peak forces in the lower back up to 5092 N during lifts and transfers. Direct measurements indicated that workers spent half to two-thirds of the day on feet, had oxygen uptakes 0.59–0.63 L/min, and mean heart rates 89–107 bpm across the workday. No study provided estimates specifically for temporary workers.

**Conclusion:**

Results suggested that eldercare work is perceived as demanding by the workers, who spend considerable time on feet, and that it comprises tasks with high spinal loads, but shows low cardiovascular demands. These findings offer a foundation for future studies exploring the short- and long-term physiological implications of occupational exposure in eldercare, including the effect of targeted interventions. Future studies are also needed that consider physical exposure differences between homecare and nursing home settings and between permanent and temporary workers, preferably using direct measurements.

**Supplementary Information:**

The online version contains supplementary material available at 10.1007/s00421-025-05962-4.

## Introduction

As global life expectancy is expected to continue to increase (Vollset et al. 2021), so too is the need for eldercare services. However, many eldercare organizations are already struggling to meet current demands, partly due to high turnover and sickness absence rates (Drennan and Ross 2019; Stone et al. 2017; Clausen et al. 2012, 2014; OECD 2020). International reports from the Organization for Economic Co-operation and Development (OECD) indicate that eldercare workers in member countries have shorter average tenures than the overall workforce, with two-thirds of OECD countries identifying worker retention in eldercare as a top policy challenge (OECD 2020). Poor working conditions, including emotional and psychological stress, high workloads (Drennan and Ross 2019) and physically demanding tasks (Clausen et al. 2014) have all been identified as contributors to worker turnover. Large cohort studies have demonstrated associations between, on the one hand, physical demands, including manual handling tasks such as lifting and transferring residents, repetitive movements, and prolonged time on feet, and, on the other, musculoskeletal disorders, pain, and increased sickness absence (Andersen et al. 2012a, 2012b, 2012c, 2013; Holtermann et al. 2013; Januario et al. 2021). Physical demands in eldercare likely have physiological implications, affecting cardiovascular load and neuromuscular fatigue, and they likely influence recovery both during and between shifts. Thus, they eventually have a potential impact on staffing shortages. Understanding these effects is critical for informing interventions based on applied physiology and thus designing of effective prevention strategies.

The eldercare workforce is made up of both licensed professionals (e.g., nurses, physiotherapists, and occupational therapists) and unlicensed workers (e.g., assistant nurses and nurse’s aides). Unlicensed workers make up the majority of the eldercare workforce (Qian et al. 2014), and provide most of the direct care, such as personal hygiene, dressing, feeding, and mobility assistance, while also offering companionship and emotional support to the elderly (Tuinman et al. 2016). Within the eldercare workforce, a substantial proportion is employed on a temporary basis. For example, in Sweden, national statistics indicate that approximately 75% of eldercare workers hold permanent positions, while around 25% are temporary workers (Swedish Association of Local Authorities and Regions (2021a); Swedish Agency for Health and Care Services Analysis (2021)). Thus, temporary employment applies to a large segment of the workforce delivering direct care, which may have implications for working conditions and workforce stability.

Temporary employment has been associated with precarious conditions such as reduced job security, lower income, and less influence at the workplace, compared to permanent employment (Jonsson et al. 2019). Temporary workers also tend to have less education, workplace training, and experience than permanent workers (Aronsson et al. 2002). These factors may make them more vulnerable at the workplace and have led to concerns that temporary workers are at increased risk for exposure to higher physical demands at the workplace (International Labour Organisation 2016). These concerns are supported by observational studies in construction, material handling, and assembly, which found that temporary workers perceived having more physically demanding tasks than permanent workers (Roquelaure et al. 2012; Kompier et al. 2009). However, it remains unclear whether this holds true in eldercare settings.

There is a need for a better understanding of the physical demands in eldercare workers in general, as well as of the potential differences related to employment form on physical exposures, but a summary of the existing literature addressing these points is currently lacking. A review could guide the design of future interventions to reorganize work tasks, reduce demands, lower sickness absence, and ultimately improve worker retention. Therefore, the aim of this scoping review was to systematically summarize the literature on physical demands in eldercare work, with particular attention to studies examining differences between temporary and permanently employed workers. We also aim to identify gaps that could inform future research on the physiological consequences of these demands, their variation across employment types and settings, and their consequences for future intervention strategies.

## Methods

We conducted a scoping review following the guidelines of the Preferred Reporting Items for Systematic Reviews and Meta-Analyses extension for Scoping Reviews (PRISMA-ScR) (Tricco et al. 2018; Munn et al. 2018).

### Search string

In January 2022, preliminary iterative searches were carried out in Scopus to test our search string. We performed a full search and screening in March 2022, followed by an updated search in April 2024. To ensure comprehensiveness, we searched Scopus, Web of Science, and PubMed as they together cover a broad range of disciplines relevant to eldercare, including health sciences, occupational medicine, and social sciences. The review focused on peer-reviewed original research to ensure methodological quality; gray literature was not included. The search strings were tailored to each database and limited to titles, abstracts, and keywords (TITLE-ABS-KEY). Our final search string is available in Supplementary Table 1.

### Inclusion and exclusion process

We considered original, full-length scientific papers written in Swedish or English and published in peer-reviewed journals. To be included, studies needed to present original data collected on workers in eldercare settings, such as homecare or nursing homes, excluding hospice, hospital care, or unpaid family caregiving. To maintain the focus on the demands experienced by eldercare workers and secure a reasonable size of the study population, we required that at least 70% of the workers—according to explicit data reported in the publication or to our best judgment—had to be involved in direct care of aged clients, and that results were reported for a minimum of 20 workers.

**Fig. 1:**
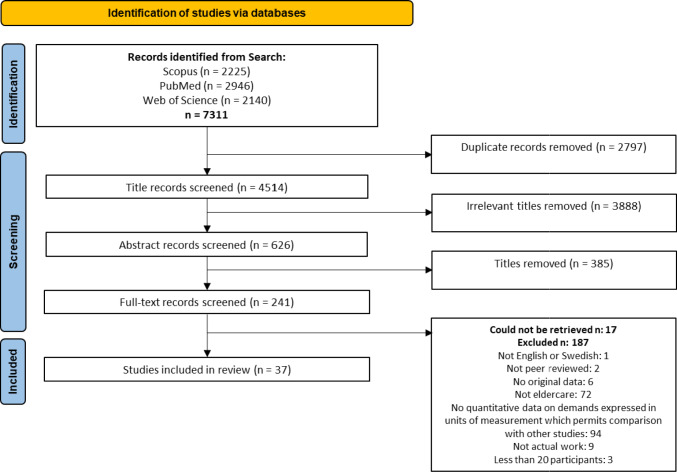
Identification and selection process of the review

Studies were included if they quantitatively assessed occupational physical demands in samples with *n* ≥ 20 and presented results in units of measurement which permitted comparison with other studies, such as compression or shear forces in N, time in different physical behaviors (e.g., percent working time or percent wake time), or physiological measures, such as heart rate in beats per minutes. We included studies based on self-reported data if they used specific indices or rating scales for physical demands, such as Borg’s Rating of Perceived Exertion (Borg 1998), but we excluded studies using broader measures of demands, like the quantitative demands index in the Copenhagen Psychosocial Questionnaire (Berthelsen et al. 2020). Qualitative data (e.g., based on interviews) were excluded. Exclusions were based on a hierarchical list of exclusion criteria, reflecting (violations of) our inclusion criteria (Fig. 1).

### Screening process

Our search identified a total of 7311 titles, reduced to 4514 titles after removal of duplicates (Fig. 1). The screening process began with an initial screening of titles, followed by a screening of abstracts, and then full texts. NL performed the initial title screening and excluded papers that were determined to not be relevant for the review, resulting in the exclusion of 3888 titles. The remaining 626 abstracts were randomly assigned in equal numbers to pairs of the co-authors who independently screened their assigned titles in Rayyan (Ouzzani et al. 2016). Any disagreement between the two reviewers in a pair was first discussed within the pair. If the pair could not come to an agreement, a third reviewer was brought in for a final vote. A total of 241 titles advanced to the full-text screening, which was performed in the same way as the abstract screening. The full-text screening resulted in 37 articles for inclusion in the review. No further searching, including forward and backward citation searches, were conducted beyond the original database searches.

We extracted information on the measurement instruments used in these 37 studies, the types of data collected, as well as descriptive statistics for the reported physical demands, including point estimates (e.g., mean, median, or mode) and measures of dispersion (e.g., standard deviations, interquartile ranges).

### Risk of bias assessment

Risk of bias (RoB) was assessed independently by pairs of reviewers using a modified version of the Joanna Briggs Institute’s (JBI) quality checklist for prevalence studies (Munn et al. 2015) (see supplement for our modifications) and followed the same conflict resolution process as in the screening. The checklist consists of nine questions to assess the overall quality of individual articles. In the JBI checklist, each question has four response options: *Yes*, *No*, *Unclear*, and *Not applicable*. In our modified version, we only used two response options: *Yes* or *No*, and included *Unclear*, and *Not applicable* in the *No* category to have a conservative quality assessment approach. This decision was made to avoid overestimating the quality of studies showing insufficiencies in reporting or lacking information regarding certain criteria. A *Yes* received one point, and a *No* received zero points. The maximal score was nine points.

### Study characteristics

The 37 included articles were published between 1989 and 2023 and reported data from 25 distinct cohorts. Population sizes ranged from 20 (the lower limit set by us) to 7025 workers. Participants were predominantly female (71–100% of each study population) and the mean study population age ranged from 29.5 to 53.3 years. Most studies were conducted in Denmark (*n* = 12), followed by the United States (*n* = 10), Sweden (*n* = 5), Hong Kong (*n* = 3), Taiwan (*n* = 2), Canada (*n* = 1), Finland (*n* = 1), Japan (*n* = 1), New Zealand (*n* = 1), and Norway (*n* = 1). Twenty-four studies assessed workers in nursing homes, 7 in homecare, and 6 covered both settings. Almost all studies which included both homecare and nursing home workers presented findings combining workers from both settings (i.e., without considering or comparing the physical demands based on workplace). The study populations included licensed workers, such as registered nurses and physiotherapists, and unlicensed workers, such as assistant nurses and nurse’s aides.

None of the studies included in the review reported the physical demands specifically for temporary workers.

The included studies employed a wide range of methods to measure physical demands. We grouped the studies into three method categories: self-reports, where workers rated their perception of the demands (*n* = 23); biomechanical models, where researchers estimated demands using a biomechanical model based on observation, for example spinal loads estimated from postures (*n* = 6); and direct technical measurements, where demands were directly assessed using technical measurement, for example, by accelerometers, heart rate monitors, or indirect calorimetry (*n* = 8). Eight studies used multiple approaches and were classified based on the following order of priority: direct measurement, biomechanical model, and self-report. For instance, if a study used both direct measurements and self-reports, it was classified as direct measurement; if it used both biomechanical models and self-reports, it was classified as biomechanical model.

### Risk of bias

The RoB showed some common quality issues across all studies, including a lack of detail in how the sample relates to the target population (criterion 1), sampling strategies (criterion 2), and descriptions of the study setting (criterion 4) (Supplementary Table 2). In addition, several studies lacked clear information on sample coverage, i.e., whether all subgroups within the sample participated to the same extent (criterion 9) (Tables 1, 2, and 3).

**Table 1:** Studies using self-reported measurements to assess physical demands

Author	Year	Country	Care setting	Aim	N	Age (mean ± SD)	Percent women	Measurements	Results	ROB (0–9)
Cheung et al. (2018)	2018	Hong Kong	Nursing home	To determine the extent of work-related musculoskeletal symptoms in nursing assistants, and the factors associated with them	387	51.1 ± 9.6	98%	Borg’s RPE (0–10) Ergonomic exposures (scale range NA)	The total study population rated their perceived exertion as 5.1 ± 2.2. Workers with musculoskeletal disorders indicated higher frequencies of ergonomic exposures (15.0 ± 7.9) compared to their symptom-free counterparts (11.3 ± 8.4)	6
Cheung et al. (2021)	2021	Hong Kong	Nursing home	To identify associated risk factors for work-related musculoskeletal symptoms among nursing assistants in nursing homes	440	51.1 ± 9.6	98%	Ergonomic exposures (scale range NA)	Workers with musculoskeletal disorders indicated higher frequencies of ergonomic exposures (16.3 ± 7.4) compared to their symptom-free colleagues (12.8 ± 8.41)	3
Clausen et al. (2013)	2013	Denmark	Mixed	To investigate whether psychosocial working conditions predict the development of low back pain in female eldercare workers while adjusting for physical workload and depressive symptoms	1537	46.3 ± 9.1	100%	Hollman Index (0–35)	Pain-free workers rated their physical demands as 16.5 ± 9.0 on average	6
Clausen et al. (2014)	2014	Denmark	Mixed	To investigate reasons for actual turnover among eldercare staff and to investigate changes in job design that could prevent turnover	7025	Workers who quit = 40 ± 10 Workers who retired = 60 ± 2.9 Workers who stayed = 46.3 ± 8.9	Workers who quit = 95.2% Workers who retired = 94.7% Workers who stayed = 96.3%	Physical workload (0–100)	Employees who quit their job early rated the physical workload higher (48.4 ± 22.6) compared both to employees who retired from the job (42.7 ± 20.8) and those who stayed in the job (45.1 ± 21)	5
Dill et al. (2013)	2013	The U.S.	Nursing home	To examine the relationship between job satisfaction, intention, and retention of nursing assistants in nursing homes and the role that “contingency factors” play in employment intentions and retention	315	NA	NA	Physical workload (1–4)	The workers perceived their physical exhaustion as 2.7 ± 0.59 following a shift	3
Feng et al. ((Feng, et al., 2007))	2007	Taiwan	Nursing home	This study aimed to assess the prevalence of and risk factors for different measures of lower back pain among nursing assistants in Taiwan	244	43.3 ± 7.9	100%	Borg‘s RPE (0–14)	On average, nursing aides rated their perceived exertion at 9.1 ± 2.1 on a 14-point scale, with 15.6% of the study sample rating above 10, which was defined as exertion	4
Gold et al. (2018)	2018	The U.S.	Nursing home	The goal of this study was to identify work-related and personal factors associated with knee pain among nursing home employees both cross-sectionally and prospectively	2643	41.6 ± 13.1	82%	Composite physical exposure (5–20)	The average composite score for physical demands were 11.7 ± 3.6	4
Gold et al. (2017)	2017	The U.S.	Nursing home	To examine whether there was a reduction in low back pain prevalence and risk following safe resident handling program implementation, concurrent with the effect on workers’ compensation claim rates. To examine whether LBP prevalence and risk following SRHP implementation were associated with self-reported lift device usage, other occupational exposures and/or health behaviors	1154	41.1 ± 13.1	91%	Composite physical exposure (5–20)	The average composite score for physical demands were 11.9 ± 3.5	3
Gonge et al. (2001)	2001	Denmark	Mixed	The aim of this study was to investigate psychosocial factors and physical exertion at work in relation to the onset of low back pain	157	44.5 ± 9.5	100%	Borg’s RPE (0–14)	The average rating of perceived exertion was 5.6 ± 2.5	5
Horneij et al. ( 2001)	2001	Sweden	Homecare	The objectives of the present study were to evaluate and compare the effects of two different intervention programs in working homecare personnel on: (1) reported neck, shoulder and back pain, (2) intermediate indicators such as perceived physical exertion at work and perceived work-related psychosocial factors	62	44 (27–60)*	100%	Borg’s RPE (6–20)	The average rating of perceived exertion was 14.5 ± 1.9	4
Hsieh et al. (2022)	2022	Taiwan	Nursing home	The purpose of this study is to understand the work content of home caregivers and the prevalence of musculoskeletal disorders in nine body parts and investigate the correlation between the work content and musculoskeletal disorders	149	50 ± 9.8	94%	Effort (0–10)	The highest effort levels were observed for transfer of toilet and wheelchair (6.9 ± 1.8) followed by transfer of bed and wheelchair (6.1 ± 1.4). Other tasks, like assisting during baths (5.1 ± 1.9) and passive range of motions exercises (5.1 ± 2.0) were similar	5
Januario et al. (2019)	2019	Denmark	Nursing home	The aim of this multilevel study is to investigate the extent to which psychosocial working conditions are associated with the physical exertion of eldercare workers	536	45 ± 10.9	95%	Borg’s RPE (0–10)	On average, the workers rated their physical exertion as 6.8 ± 2.0	4
Jensen et al. ( 2006)	2006	Denmark	Homecare	The objective of the present study is to evaluate the effectiveness of a transfer technique intervention and stress management intervention in reducing lower back pain in a group of eldercare workers	61	44 ± 8.5	100%	Physical workload (1–7)	The average rating of perceived exertion was 5.4 ± 2.4	7
Jensen et al. (2011)	2011	Denmark	Nursing home	To explore whether collective efficacy as a social contextual factor has a moderating effect by providing group members with means to cope with their perceived stressors. More specifically, we investigate whether collective efficacy moderates the associations between physical workload and intention to leave and sickness absence	6791	46 ± 10.1	100%	Physical workload (1–7)	The average rating of perceived exertion was 3.9 ± 1.3	5
Karstad et al. ( 2018)	2018	Denmark	Nursing homes	To investigate longitudinal associations between physical and psychosocial working conditions and occurrence of musculoskeletal disorders and its consequences (pain-related interference with daily work activities and sickness absence) among Danish eldercare workers	553	45.7 ± 10.9	95%	Borg’s RPE (0–10)	On average, the workers rated their perceived exertion as 6.6 ± 1.9	5
Larsson et al. (2012)	2012	Sweden	Homecare	The aim of this research was to identify factors promoting work ability and self-efficacy in care aides and assistant nurses within homecare services	137	Assistant nurses = 46.5 ± 9.3 Care aides = 44 ± 12.6	Assistant nurses = 94% Care aides = 91%	Borg’s RPE (6–20)	On average, assistant nurses rated their perceived exertion as 13.2 ± 2.3, and care aides rated theirs as 13.3 ± 2.6	4
Larsson et al. (2013)	2013	Sweden	Homecare	This study aims to examine homecare workers’ perceptions of health, risks, working conditions, and risk management within their organization	133	45.3 ± 10.8	92%	Borg’s RPE (6–20)	On average, respondents rated the perceived physical exertion as 13.2 ± 2.4, and 37% of them perceived their job to be highly physically demanding	3
Owen et al. (1999)	1999	The U.S.	Nursing home	The primary goal of this study was to compare the perceived physical exertion experienced by the nursing staff while transferring residents from bed to chair and chair to bed using an ergonomically designed transfer technique versus traditional methods of transfer (via gait belt or mechanical lift)	448	NA	NA	Borg’s RPE (0–10)	Employee ratings for transfers from bed to wheelchair averaged 5.6 ± 1.7, whereas transfers from wheelchair to bed were rated at 5.1 ± 1.8	1
Owen et al. (2003)	2003	The U.S.	Homecare	There were three purposes of this study: 1. Determine the perceived physical stressfulness of homecare tasks. 2. Identify what home health aides and nurse observers thought contributed to making these tasks stressful. 3. Develop ideas for reducing back stress applicable to all homecare workers	33	37 ± 12.2	100%	Borg’s RPE (0–10)	Lifting patients manually in bed received the highest rating for lower back physical demand (9.2 ± 0.7), followed by putting on anti-embolism stockings (9.1 ± 0.7), transferring from chair to chair (8.8 ± 1.2), giving tub baths (8.2 ± 1.5), and repositioning in a chair (8.2 ± 1.3)	3
Rasmussen et al. (2016)	2016	Denmark	Mixed	To evaluate whether the multi-faceted intervention with participatory ergonomics, physical training and cognitive behavioral training was effective for the intermediate outcomes physical exertion, occupational lifting, muscle strength, fear avoidance beliefs and support from management and for the distal outcomes work ability and sickness absence due to low back pain	594	47 ± 10.2	93%	Borg’s RPE (0–10)	The average rating of physical exertion was 6.4 ± 2.2	4
Rasmussen et al. (2015)	2015	Denmark	Mixed	The objective of this prospective cohort study was to investigate whether self-reported physical capacity, that is, aerobic fitness, balance, muscle strength, flexibility, and overall physical capacity, predicted risk for register-based long-term sickness absence among female healthcare workers in a 1-year follow-up period	4736	High physical capacity = 46 ± 9 Medium physical capacity = 45 ± 10 Low physical capacity = 45 ± 10	100%	Hollman index (0–35)	The employees were categorized into three groups based on their physical capacity levels: low, medium, and high. The group with lower physical capacity rated their physical workload as 20 ± 10, the medium group as 20 ± 9.8, and the high-capacity group as 19 ± 10.2	6
Takahashi et al. (2006)	2006	Japan	Nursing home	The purpose of this study was to investigate the relationship between musculoskeletal disorders and sleep problems in a sample of employees working in nursing homes	98	33.4 ± 9.6	81%	Physical workload (0–4)	Workers who reported both arm pain and daytime sleepiness rated the physical workload on the lower back as higher (3.0 ± 1.5) when transferring patients from wheelchair to bathtub, compared to those with arm pain but no daytime sleepiness (2.1 ± 1.5), those without arm pain but with daytime sleepiness (1.5 ± 1.0), and those without both arm pain and daytime sleepiness (1.6 ± 1.5). Similar patterns were observed for most ratings of physical workload	3
Yeung et al. (2012)	2012	Hong Kong	Nursing home	The purpose of this study is to use a multifactorial approach to identify the possible risk factors that might contribute to the occurrence of low back pain among personal care workers in an old age home	36	42.2 ± 5.3	NA	Physical workload (1–7)	Workers perceived walking (6.9 ± 0.4), repetitive activities involving the back (6.7 ± 0.6), standing (6.9 ± 0.4), repetitive activities involving the lower limbs (6.7 ± 0.6), fixed force load on the upper limb (6.6 ± 0.8), lifting and carrying heavy objects (6.3 ± 1), and assisting patients in and out of bed (6.5 ± 1.3) as the most physically demanding tasks	2

*ROB*, risk of bias; *, range instead of SD; *NA*, not available

**Table 2:** Studies using biomechanical models to assess physical demands

Author	Year	Country	Care setting	Aim	N	Age (mean ± SD)	Percent women	Measurements	Results	ROB (0–9)
Garg et al. (1992)	1992	The U.S.	Nursing home	A prospective epidemiologic study was conducted in two units of a nursing home to demonstrate the efficacy of an ergonomic intervention strategy to reduce back stress to nursing personnel	38	32 ± 10.5	95%	Compressive forces Borg’s RPE (6–20)	The study assessed the rating of perceived exertion during 18 common patient handling tasks and estimated compressive forces on the L5/S1 disc during manual lifting. Perceived exertion for the tasks ranged between 11.3 and 14.1. Changing attends while patients were in a wheelchair was identified as the least exerting task, whereas transferring patients from the toilet to the wheelchair was ranked as the most exerting. The estimated compressive forces during manual lifting were measured at 4751 ± 106	3
Garg et al. (1992)	1992	The U.S.	Nursing home	The objective of this study was to perform an ergonomic evaluation of patient handling tasks. In particular, the objective was to identify patient handling tasks perceived to be most stressful by nursing assistants and perform a biomechanical evaluation of these tasks	38	32 ± 10.5	95%	Compressive forces Hand forces Joint angles Rotation angles Borg’s RPE (6–20)	The study assessed the rating of perceived exertion (RPE) during 18 common patient handling tasks and estimated compressive forces on the L5/S1 disc during manual lifting. Perceived exertion for the tasks ranged between 11.3 and 14.1. Changing attends while patients were in a wheelchair was identified as the least exerting task, whereas transferring patients from the toilet to the wheelchair was ranked as the most exerting. The estimated compressive forces during manual lifting were measured at 4751 ± 106	3
Holmes et al. (2010)	2010	Canada	Nursing home	The purpose of this study was to evaluate peak and cumulative lumbar spine loads experienced by personal support workers	20	47.2 ± 9.4	100%	Compressive forces Lateral shear forces Anterior–posterior shear forces	The peak joint compression force (N) at L4/L5 during single-person manual lifting was measured at 5092 ± 500.1. For manual lifts involving two workers, the peak compression force was 3163.6 ± 425.6. Other activities generated varying peak compression forces: manually adjusting patients in bed (3244.1 ± 393.6), dressing patients (2120 ± 232.8), and walking and pushing patients in wheelchairs (1242.1 ± 264.8 and 1301.3 ± 224.1, respectively)	3
Kurowski et al. (2014)	2014	The U.S.	Nursing home	To obtain a comprehensive analysis of the physical workload of clinical staff in long-term care facilities, before and after a safe resident handling program	63	NA	75%	Compressive forces Joint angles	The estimates of average compressive forces (N) on L5/S1 during tasks with moderate trunk flexion (20–45°) were 1410 N, increasing to 1980 N during severe flexion (> 45°). The physical workload index was 30–40% higher compared to that of registered nurses	2
Owen et al. (1992)	1992	The U.S.	Nursing home	The purpose of this paper is to describe four methods of identifying stressful work tasks, and compare results obtained with each method based on experiences using each method in one nursing home	38	32.8 ± 10.8	95%	Compressive forces Borg’s RPE (6–20)	The average estimated compressive forces (N) at L5/S1 for these tasks were as follows: 4810 N for toilet to wheelchair, 3676 N for wheelchair to toilet, 4877 N for wheelchair to bed, and 3991 N for bed to wheelchair. The four most physically demanding work tasks, as reported by participants, included transferring patients from the toilet to the wheelchair, from the wheelchair to the toilet, from the wheelchair to the bed, and from the bed to the wheelchair. The rating of perceived exertion for these tasks was 12.7 ± 3.1, 12.3 ± 2.8, 12.2 ± 3.2, and 11.9 ± 3.0, respectively	2
Owen et al. (1991)	1991	The U.S.	Nursing home	The purpose of this study was to reduce back stress for nursing personnel by changing the physical job demands	38	32.8 ± 10.8	95%	Compressive forces Borg’s RPE (6–20)	The estimated compressive forces (N) on the L5/S1 during manual lifting of patients were 4795 ± 183. In addition, when transferring a patient of median weight from the wheelchair to the toilet, the compressive forces were measured at 4800. The workers perceived transferring clients from the toilet to the wheelchair as the most physically demanding task, followed transferring clients from the wheelchair to the toilet. The third-ranked task involved moving patients from the chair to the bed, while returning them from the bed to the chair was ranked fourth. Participants reported the highest perceived exertion in the lower back when transferring patients from the toilet to the wheelchair (14.3 ± 2.7)	3

*ROB*, risk of bias; *, range instead of SD; *NA*, not available

**Table 3:** Studies using direct measurements to assess physical demands

Author	Year	Country	Care setting	Aim	N	Age (mean ± SD)	Percent women	Measurements	Results	ROB (0–9)
Januario et al. (2020)	2020	Denmark	Nursing home	This study aims at determining the extent to which the composition of physical behaviors at work is associated with perceived physical exertion among eldercare workers at nursing homes, and the extent to which these associations are modified by psychosocial resources at work	399	46.2 ± 10.5	100%	Accelerometer measured physical activity Borg’s RPE (0–10)	The workers spent 37.4 ± 12.5% of the working time sitting, 32.5 ± 7.8% standing, 16.1 ± 4.8% doing light activity, and 14 ± 3.9% doing moderate activity, and rated their perceived exertion as 6.9 ± 1.9	5
Ljungberg et al. (1989)	1989	Sweden	Nursing home	The aim of this study was accurately to quantify lifting and carrying work as well as the physical workload in two occupations with a high frequency of back disorders	24	29.5 ± 11	100%	Accumulated load Number of steps per work hour Oxygen uptake Ventilation rate Heart rate Borg’s RPE (6–20)	The nursing aides experienced an average lifting load of 2270 ± 1007 N. In addition, they took an average of 2202 ± 676 steps per hour. Their average oxygen uptake was 0.59 ± 0.10 L/min, with a pulmonary ventilation of 17.8 ± 2.5 L/min. Furthermore, the nursing aides maintained an average heart rate of 100 ± 10 beats per minute. Perceived exertion ranged between 8.5 and 10.5 depending on task	3
Mänttäri et al. (2023)	2022	Finland	Homecare	To investigate the physical workload of homecare nurses and to determine whether the intensity of physical work strain has an impact on overall recovery from work measured by heart rate variability	95	43.7 ± 12.0	92%	Heart rate Heart rate variability	The average heart rate recorded during shifts was 89.1 ± 11.7, and the average heart rate variability measured as the root mean square of successive differences between normal heartbeats and expressed in milliseconds (ms) was 27.8 ± 11.3 ms. The lowest variability was observed during the workday (20.0 ± 9.3 ms) and greatest during sleep (37.0 ± 17.7 ms)	6
Neupane et al. (2020)	2020	Denmark	Nursing homes	To explore the prospective association of objectively measured and self-reported occupational physical activity with multisite musculoskeletal pain among Danish eldercare workers	389	46.3 ± 10.5	100%	Accelerometer measured physical activity	Objectively measured occupational physical activity was calculated as the sum of cycling, running, stairclimbing, and walking activities at work per day. On average, employees achieved 1.16 ± 0.36 h of physical activity during the working day	7
Ribeiro et al. ( 2011)	2011	New Zealand	Nursing home	The aim of the present study is to examine the within-day reliability of the Spine angle for monitoring daily posture and to develop preliminary estimates of cumulative postural exposure of health care workers at a residential home for the elderly	21	46.3 ± 15	95%	Accelerometer measured trunk postures and trunk movements	On average, workers spent 5% of their work time in forward flexion postures exceeding 30°, and 3.0% of their work time in backward bending postures exceeding 30°. Approximately 0.2% of the total recorded time was dedicated to postures exceeding 60° of lumbo-pelvic forward flexion. The threshold for 30° of forward flexion was surpassed 1069 ± 2157.1 times per hour, for 45° an average of 121.5 ± 223.7 times per hour, and an average of 8 ± 21.8 times per hour for 60°	5
Stevens et al. (2021)	2021	Denmark	Nursing home	The aims of the present study were to: (i) evaluate how much of eldercare workers’ total variance in step-rate occurs at different organizational levels (i.e., between shifts, workers, wards, and nursing homes) and (ii) identify determinants of step-rate at each of these different levels	1287	46 ± 10.6	95%	Accelerometer measured steps/h	On average, workers took 1124 ± 315 steps/h at work	6
Tjøsvoll et al. (2022)	2022	Norway	Homecare	To assess physical exposures in homecare workers in a large Norwegian municipality using wearable sensors	114	36.7 ± 12.4	71%	Accelerometer measured occupational physical activity Heart rate reserve	Workers sat for most of the day (50%). Occupational physical activity included standing (25.2%), moving (11.4%), walking slow (1.9%), walking fast (8.3%), running (0.05%), stair climbing (1.2%), and cycling (0.3%). The average %heart rate reserve was calculated and found to be highest for cycling (48.5%), running, (39.3%), stair climbing (38.4%), and walking fast (37.2%). The lowest % heart rate reserve was measured for sitting (22.9%)	6
Torgén et al. (1995)	1995	Sweden	Mixed	The primary aim of this study was to investigate physical workload, physical capacity, physical strain and perceived health among female aides aged over 45 years in municipal homecare service. A second aim was to compare the workload and strain between the two main types of homecare service available in Sweden today	20	53.3 ± 5.2	100%	Oxygen uptake Heart rate Joint angles Number of steps per shift Borg’s RPE (6–20)	The average heart rate observed was 96 beats per minute, equivalent to 25% of the heart rate reserve during work. Cleaning tasks were associated with the highest cardiovascular load, with a heart rate of 107 beats per minute, representing 34% of the heart rate reserve. The mean oxygen consumption during work was measured at 0.63 L/min, and, on average, workers took 6172 steps throughout the workday. In addition, the rating of perceived exertion was assessed repeatedly over the workday, revealing that as the workday progressed, workers consistently reported higher levels of exertion	1

### Self-reported physical demands

Self-reported data indicated moderate to high physical demands, with average ratings ranging from 40 to 98% of the maximal scale scores across studies, corresponding to moderate to high values on the respective measurement scales (Table 1). Tasks such as patient lifts and transfers, and repetitive movements were perceived as the most physically demanding (Hsieh et al. 2022; Owen and Fragala 1999; Owen and Staehler 2003; Yeung 2012), and workers reported the highest demands for the lower back, followed by the upper back and shoulders (Owen and Fragala 1999; Owen et al. 1992). The most common instrument used to collect self-reported physical demands was modified versions of Borg’s perceived exertion scale (Borg 1998), which was used in 18 studies (Tables 1, 2, 3), which was used in 18 studies (Tables 1, 2, 3), with variations in the numerical scale between studies (e.g., 0–10, 1–7, or 6–20), anchor labels, and the context of the ratings. Participants were asked to rate their perceived exertion for different scenarios, such as the whole workday, specific tasks, particular body parts, or various times throughout the workday. Seven studies employed indices that combined different aspects of physical demands, such as posture and load or movement speed, into a single number representing an overall demand (Clausen et al. 2013, 2014; Cheung et al. 2021; Dill et al. 2013; Gold et al. 2017, 2018; Rasmussen et al. 2015).

### Biomechanically modeled physical demands

Six studies used biomechanical models (Table 2) to estimate physical demands, including intervertebral lumbar loads during commonly performed work tasks, such as, dressing, repositioning, and transferring of patients. Modeling studies found peak forces at the L5/S1 disc of the lower back between 4751 and 5092 N during lifts and transfers (Owen et al. 1992; Garg and Owen 1992; Garg et al. 1992; Holmes et al. 2010; Owen and Garg 1991), 3244 N during repositioning patients in bed (Holmes et al. 2010), and between 3991 and 4877 N during transfers to and from wheelchairs (Owen et al. 1992). Studies that combined model estimates with ratings of perceived exertion consistently demonstrated that work tasks with higher compression forces, like patient transfers between the bed and toilet, were associated with higher ratings of perceived exertion (Owen et al. 1992; Garg and Owen 1992; Garg et al. 1992).

### Technical measurement of physical demands

Eight studies measured physical demands using direct technical methods (Table 3). Four of these studies used accelerometers to assess specific aspects of occupational physical activity, such as time spent sitting, light and moderate-intensity activity, and daily step counts (Januario et al. 2020; Neupane et al. 2020; Stevens et al. 2021; Tjøsvoll et al. 2022). Four studies evaluated physiological demands, including oxygen uptake and ventilation rate (Jungberg et al. 1989; Torgén et al. 1995), as well as heart rate indices (Tjøsvoll et al. 2022; Jungberg et al. 1989; Torgén et al. 1995; Mänttäri et al. 2023). Together, the results suggested that eldercare workers spent half to two-thirds of the working time on their feet (Januario et al. 2020; Tjøsvoll et al. 2022), took around 1000 steps per hour (Stevens et al. 2021), and accumulated a total of 6000–7000 steps during a shift (Tjøsvoll et al. 2022; Torgén et al. 1995). Estimates of cardiovascular demands showed oxygen uptakes between 0.59 and 0.63 L/min (Jungberg et al. 1989; Torgén et al. 1995) and mean heart rates between 89 and 107 bpm during working hours (Jungberg et al. 1989; Torgén et al. 1995; Mänttäri et al. 2023). Expressing work intensity as a percentage of heart rate reserve (%HRR), one study reported that cleaning and running errands were associated with the highest intensities. The median time spent on these tasks accounted for 7% and 13% of the workday, respectively (Torgén et al. 1995). Another study combined physical behaviors with %HRR and found that sitting and standing constituted 49.6% and 25.2% of the workday, corresponding to %HRRs of 22.9% and 32.7%, respectively. Cycling, though associated with a %HRR as high as 48.5%, accounted for only 0.3% of the workday (Tjøsvoll et al. 2022). In summary, the technical measurements showed that eldercare work was characterized by prolonged time on feet, but that the physical activity was of low in terms of cardiovascular intensity.

## Discussion

This review summarized the literature on occupational physical demands in eldercare workers and considered, to the extent possible, whether demands differed between permanent and temporary workers. To our knowledge, this is the first comprehensive review to address both perceived and objectively measured biomechanical demands in eldercare workers, with implications for physiological outcomes. Relying on a systematic search strategy, we found 37 studies from ten different countries which met our inclusion criteria. Overall, the studies showed that eldercare workers perceived their work as physically demanding and that common work tasks, such as lifts and transfers, led to high compressive forces on the spine. The few studies that used direct measurement indicated that workers spent a considerable time of the working day on their feet, but that average daily cardiovascular loads were low. None of the studies presented data on physical demands specifically for temporary eldercare workers.

The findings from this review may have practical implications that can guide the organization of eldercare work, including task allocation to workers and the use of assistive devices. The consistent reports of high perceived physical demands and substantial time spent on feet suggest that work could be organized to allow for more task variation and more opportunities for periods of sitting during work hours. For example, tasks may be identified that can be performed seated, and the organization of tasks may support recovery during longer periods of work otherwise involving prolonged standing or manual handling. Results also indicate that assistive devices may reduce biomechanical loads during transfers and lifts (Garg and Owen 1992; Garg et al. 1992; Owen and Garg 1991); however, the employer must ensure that these devices are integrated and consistently used in daily workflows to help reduce perceived exertion (Noble and Sweeney 2018; Kyriakidis et al. 2021).

Our RoB assessment found that many studies did not provide clear details about the data sampling methods or how their samples related to the target population, making it hard to assess the potential effects of convenience samples and self-selection on the results. In addition, many studies lacked details about the broader eldercare context, hampering interpretation of results. For example, factors like higher resident-to-staff ratios and greater caregiver dependency have been linked to increased physical demands and more frequent manual handling tasks in nursing homes (Januario et al. 2021; Kyriakidis et al. 2021; Stevens et al. 2022), and such contextual factors may account for differences in results between studies.

The included studies varied in design, assessment methods, and reported outcomes, further complicating efforts to compare and synthesize the results. We grouped the studies in three categories: self-report (*n* = 23), biomechanical modeling (*n* = 6), and direct measurement (*n* = 8). However, the categories were heterogenous, and even studies using the same measurement instrument showed differences in contexts, how the instruments were constructed, and timelines, with some asking for general ratings over whole days and others collecting repeated ratings throughout the day. For example, studies employing Borg’s scale of perceived exertion differed in their focus—asking participants to rate general workdays, specific tasks, or body parts—and in the instrument design, including prompts, anchor labels, and rating scales. This heterogeneity limits the generalizability of individual studies and reduces the ability to draw firm conclusions across studies (Schwarz 1999). To improve comparability and, thus, evidence synthesis, future studies should aim for standardized measurement protocols, including consistent use of validated scales for perceived exertion, harmonized definitions of physical behaviors, and standardized approaches to physiological monitoring.

One of our main aims was to identify studies examining differences between temporary and permanently employed workers, as temporary workers in other occupations have been observed to experience higher physical demands than the permanent staff (Roquelaure et al. 2012; Kompier et al. 2009). However, we found no studies specifically addressing this issue in eldercare, highlighting a gap in the literature.

Handling tasks, such as lifting and transferring residents, were consistently rated as the most physically demanding (Owen et al. 1992; Garg and Owen 1992; Garg et al. 1992); these tasks also resulted in high lumbar compression forces (Owen et al. 1992; Garg and Owen 1992; Garg et al. 1992). These studies showed that lifting aids and other assistive devices effectively reduced both perceived physical demands and compression forces, which have led to adoption of assistive devices and no-lift policies in many organizations. Despite this, our review suggests that perceived exertion has remained high over the 30-year period covered by the included studies, highlighting a persistent challenge of reducing physical demands in eldercare. Some research indicates that lifting aids and assistive devices may not be consistently used for all manual handling tasks (Karstad et al. 2022). However, factors beyond handling tasks may also contribute to the perception of physical demands. For instance, organizational-level factors may influence physical demands. Better workplace resources, such as more influence and higher quality of leadership, have been associated with reduced perceived exertion (Januario et al. 2019), while higher quantitative demands and work pace have been linked to increased perceived exertion (Januario et al. 2019, 2020). Studies in the review indicated that workers spent half to two-thirds of their workday on their feet (Januario et al. 2020; Tjøsvoll et al. 2022), which may also contribute to high perceived physical demands, although none of the studies addressed this association. Nevertheless, extensive time on feet has been associated with fatigue and discomfort (Yeung 2012; Waters and Dick 2015) as well as musculoskeletal disorders (Coenen et al. 2017, 2018) and may play a role for the sustained high perceived physical demands over the past 3 decades. Thus, further studies should address time on feet to reduce perceived high levels of perceived physical demands in eldercare workers.

Four studies evaluated the physical demands of eldercare work in terms of cardiovascular indices (Tjøsvoll et al. 2022; Jungberg et al. 1989; Torgén et al. 1995; Mänttäri et al. 2023), and they indicated that the observed cardiovascular demands were consistent with low-intensity activities, such as walking and everyday house chores (Almeida et al. 2018; Spruit et al. 2011), thus agreeing with the predominantly low-intensity behaviors that typically engaged the workers, such as sitting, standing, and slow walking. However, self-reported demands may differ from physiological demands. The linear relationship between heart rate or oxygen uptake and perceived exertion commonly observed during whole-body activities such as walking, running, or cycling in laboratory settings (Scherr et al. 2013; Utter et al. 2004) may not apply to eldercare work. Oxygen uptake and heart rate reflect aerobic demands (Hargreaves and Spriet 2020), but not local biomechanical demands on joints, ligaments, tendons, and muscles. These demands likely influence the perceived exertion scores reported by the workers during prolonged periods on feet, and may explain the disagreement between heart rate or oxygen uptake measurements and self-reported exertion observed in the reviewed studies.

This highlights the need for integrative physiological studies to fully characterize the occupational exposure profile, combining objective measures such as heart rate, heart rate variability, and oxygen uptake, biomechanical data such as local loads on the low back, and subjective ratings of demands. Such studies could clarify associations between perceived exertion and biomechanical load patterns, including periods of recovery over time. In addition, experimental or observational studies integrating physiological monitoring, ergonomic assessments, and contextual data such as psychosocial demands would offer valuable insights into how temporal patterns of exposures affect short- and long-term health outcomes.

Six studies included workers from different eldercare settings, such as homecare and nursing homes, but comparisons of physical demands between the two were limited. Nursing homes typically accommodate individuals with greater care needs than homecare settings (Schön et al. 2016; Swedish Association of Local Authorities and Regions (2021b); Szebehely et al. 2017) and workers in nursing homes often work together with colleagues in specialized facilities, whereas homecare workers generally work alone in less customized environments (Wipfli et al. 2012). Moreover, homecare workers travel between caretaker residences during working hours, while nursing home workers typically work at one facility. All these factors may lead to differences in the distribution and accumulation of physical demands over the working day between workers in homecare and nursing homes, and future studies should examine differences between settings, and thus whether interventions in the work environment should be tailored to either sector.

While we conducted a comprehensive systematic search of the literature, several methodological limitations must be acknowledged. The study was not pre-registered; however, an explicit and systematic review process was established within the research group prior to screening articles and extracting data. While this predefined approach aimed to ensure methodological rigor, the lack of formal pre-registration may increase the risk of bias in study selection and reporting. The initial title screening was conducted by a single reviewer, which may have introduced selection bias. We did not review the reference lists of included studies, or performed a ‘cited by’ search; both initiatives might have led to additional relevant studies. Furthermore, we chose not to include psychosocial demands and resources, despite their recognized influence on health outcomes (Stone et al. 2017; Midje et al. 2022; Krsnik and Erjavec 2023). This omission may restrict the overall understanding of the challenges faced by eldercare workers. We did not have an exclusion criteria based on publication year, resulting in the inclusion of studies dating back over three decades. Although these older studies may offer valuable insights, they may not accurately reflect current eldercare practices, given that work environments and work tasks have likely evolved due to changes in healthcare policies and technological advancements. We also did not impose geographical restrictions on included studies despite known variations in labor laws, eldercare practices, and welfare systems between different countries (Laxer et al. 2016) which may influence work environment factors, and thus physical demands. Lastly, we included studies involving workers from both homecare and nursing home settings, as well as studies including both licensed and unlicensed workers. Work demands may differ between these settings and worker groups due to variations in work organization, caretaker needs, and physical work tasks.

## Conclusions

The present scoping review aimed to summarize the literature on occupational physical demands in eldercare workers and consider, to the extent possible, whether demands differed between permanent and temporary workers. The included studies suggested that eldercare workers perceive their work as physically demanding, that common work tasks like lifts and transfers were both perceived as physically demanding and generated high spinal loads, that workers spend large parts of the working day on their feet, and that cardiovascular loads during work were low. Our findings highlight the physically demanding aspects of eldercare work and can inform interventions to promote worker health and wellbeing, while also providing valuable insights for eldercare organizations seeking to identify potential risk exposures in the workforce. We could not identify any study specifically investigating the physical demands of temporary workers. In light of these findings, there is a need for future interdisciplinary studies in both homecare and nursing home settings, and among permanent as well as temporary workers that examine how the temporal pattern of biomechanical exposures translate into physiological responses and perceptions of effort in different eldercare settings and for different groups of workers. Such studies would improve our understanding of demands, fatigue, and recovery in eldercare workers and inform ergonomic and organizational interventions that are better tailored to the conditions in each care environment.

## Supplementary Information

Below is the link to the electronic supplementary material.

Supplementary file1 (DOCX 14 KB)

Supplementary file2 (DOCX 21 KB)

## Data Availability

Search strings used are available in supplementary Table 1.
