# Supplementary material for: Occupational physical demands in eldercare workers: a systematic scoping review of studies reporting quantitative data
Source: Eur J Appl Physiol. 2025 Sep 9;126(2):897–925. doi: 10.1007/s00421-025-05962-4 (PMC12948920; doi:10.1007/s00421-025-05962-4)
Supplement: Supplementary file 1 — Supplementary file1 (DOCX 14 KB) [file 421_2025_5962_MOESM1_ESM.docx]

**Supplementary Table 1**. Used search string.

| Description |  |
| --- | --- |
| Worker | "Personal care worker*" OR "Direct care worker*" OR "Elderly care worker*" OR" Elder care worker*" OR "Eldercare worker*" OR "Homecare worker*" OR "Home care worker*" OR "Domiciliary care worker*" OR "Long-term care worker*" OR "Long term care worker*" OR "Nursing home worker*" OR "Old age home worker*" OR "Nurse aide*" OR "Nurse's aide*" OR "Nursing aide" OR "Nurse assistant" OR "Nurse's assistant*" OR "Nursing assistant*" OR "Orderl*" OR "Healthcare assistant*" OR "Health care assistant*" |
|  | AND |
| Demand | "Physical load" OR "Physical strain" OR "Physical workload" OR "Physical activity" OR " Step*" OR "Step count" OR "Physical demand*" OR "Physiological strain" OR "Physiological demand*" OR "Biomechanical exposure" OR "Physical exertion" OR "Resident handling" OR "Resident transfer" OR "Lift*" OR "Physical behavio*" OR "Patient transfer" OR "Patient handling" OR "Work task*" OR "Occupational exposure" OR "Work exposure" |
