# Supplementary material for: Occupational physical demands in eldercare workers: a systematic scoping review of studies reporting quantitative data
Source: Eur J Appl Physiol. 2025 Sep 9;126(2):897–925. doi: 10.1007/s00421-025-05962-4 (PMC12948920; doi:10.1007/s00421-025-05962-4)
Supplement: Supplementary file 2 — Supplementary file2 (DOCX 21 KB) [file 421_2025_5962_MOESM2_ESM.docx]

**Supplementary Table 2**. Risk of bias assessment. Score per item and total score (of a possible maximum of 9) for all included studies.

| **Authors** | **Year** | **1. Was the sample frame appropriate to address the target population?** | **2. Were study participants sampled in an appropriate way?** | **3. Was the sample size adequate?** | **4. Were the study subjects and the setting described in detail?** | **5. Was the data analysis conducted with sufficient coverage of the identified sample?** | **6. Were valid methods used for the identification of the condition?** | **7. Was the condition measured in a standard, reliable way for all participants?** | **8. Was there appropriate statistical analysis?** | **9. Was the response rate adequate, and if not, was the low response rate managed appropriately?** | **Total score** |
| --- | --- | --- | --- | --- | --- | --- | --- | --- | --- | --- | --- |
| Cheung et. al | 2021 | 0 | 0 | 0 | 0 | 0 | 0 | 0 | 1 | 0 | 1 |
| Cheung et. al | 2018 | 1 | 1 | 0 | 0 | 1 | 1 | 1 | 1 | 0 | 6 |
| Clausen et. al | 2013 | 0 | 1 | 1 | 0 | 1 | 0 | 1 | 1 | 1 | 6 |
| Clausen et. al | 2014 | 0 | 1 | 1 | 0 | 0 | 0 | 1 | 1 | 1 | 5 |
| Dill et. al | 2013 | 0 | 0 | 0 | 0 | 0 | 0 | 1 | 1 | 1 | 3 |
| Feng et. al | 2007 | 0 | 0 | 0 | 0 | 0 | 1 | 1 | 1 | 1 | 4 |
| Garg et. al | 1992 | 0 | 0 | 0 | 1 | 0 | 1 | 0 | 1 | 0 | 3 |
| Garg et. al | 1992 | 0 | 0 | 0 | 1 | 0 | 1 | 0 | 1 | 0 | 3 |
| Gold et. al | 2018 | 0 | 0 | 0 | 1 | 1 | 1 | 0 | 1 | 0 | 4 |
| Gold et. al | 2017 | 0 | 0 | 0 | 0 | 1 | 1 | 0 | 1 | 0 | 3 |
| Gonge et. al | 2001 | 0 | 1 | 0 | 1 | 0 | 1 | 0 | 1 | 1 | 5 |
| Holmes et. al | 2010 | 0 | 0 | 0 | 1 | 0 | 1 | 0 | 1 | 0 | 3 |
| Horneij et. al | 2001 | 1 | 0 | 0 | 1 | 0 | 1 | 0 | 1 | 0 | 4 |
| Hsieh et. al | 2022 | 0 | 0 | 0 | 1 | 0 | 1 | 1 | 1 | 1 | 5 |
| Januario et. al | 2019 | 0 | 1 | 0 | 1 | 0 | 1 | 0 | 1 | 0 | 4 |
| Januario et. al | 2020 | 0 | 1 | 0 | 1 | 0 | 1 | 1 | 1 | 0 | 5 |
| Jensen et. al | 2011 | 1 | 0 | 1 | 1 | 0 | 1 | 0 | 1 | 0 | 5 |
| Jensen et. al | 2006 | 1 | 1 | 1 | 1 | 0 | 1 | 0 | 1 | 1 | 7 |
| Karstad et. al | 2018 | 0 | 1 | 0 | 1 | 0 | 1 | 0 | 1 | 1 | 5 |
| Kurowski et. al | 2014 | 0 | 0 | 0 | 0 | 0 | 1 | 1 | 0 | 0 | 2 |
| Larsson et. al | 2013 | 0 | 1 | 0 | 1 | 0 | 0 | 0 | 1 | 0 | 3 |
| Larsson et. al | 2012 | 0 | 1 | 0 | 1 | 0 | 0 | 0 | 1 | 1 | 4 |
| Ljungberg et. al | 1989 | 0 | 0 | 0 | 1 | 0 | 1 | 0 | 1 | 0 | 3 |
| Mänttäri et. al | 2022 | 1 | 0 | 0 | 1 | 1 | 1 | 1 | 1 | 0 | 6 |
| Neupane et. al | 2020 | 0 | 1 | 0 | 1 | 1 | 1 | 1 | 1 | 1 | 7 |
| Owen et. al | 1991 | 0 | 0 | 0 | 1 | 0 | 1 | 0 | 1 | 0 | 3 |
| Owen et. al | 1999 | 0 | 0 | 0 | 0 | 0 | 0 | 0 | 1 | 0 | 1 |
| Owen et. al | 1992 | 0 | 0 | 0 | 0 | 0 | 1 | 0 | 1 | 0 | 2 |
| Owen et. al | 2003 | 0 | 0 | 0 | 0 | 0 | 1 | 1 | 1 | 0 | 3 |
| Rasmussen et. al | 2015 | 1 | 1 | 1 | 0 | 0 | 1 | 0 | 1 | 1 | 6 |
| Rasmussen et. al | 2016 | 0 | 0 | 1 | 0 | 0 | 1 | 0 | 1 | 1 | 4 |
| Ribeiro et. al | 2011 | 1 | 0 | 0 | 0 | 0 | 1 | 1 | 1 | 1 | 5 |
| Stevens et. al | 2021 | 0 | 1 | 1 | 0 | 0 | 1 | 1 | 1 | 1 | 6 |
| Takahashi et. al | 2006 | 0 | 0 | 0 | 0 | 0 | 1 | 1 | 1 | 0 | 3 |
| Tjøsvoll SO et. al | 2022 | 1 | 1 | 0 | 1 | 0 | 1 | 1 | 1 | 0 | 6 |
| Torgén et. al | 1995 | 0 | 0 | 0 | 0 | 0 | 0 | 0 | 1 | 0 | 1 |
| Yeung et. al | 2012 | 0 | 0 | 0 | 0 | 0 | 1 | 1 | 0 | 0 | 2 |
